# Microorganisms: A Potential Source of Bioactive Molecules for Antioxidant Applications

**DOI:** 10.3390/molecules26041142

**Published:** 2021-02-20

**Authors:** Alka Rani, Khem Chand Saini, Felix Bast, Sanjeet Mehariya, Shashi Kant Bhatia, Roberto Lavecchia, Antonio Zuorro

**Affiliations:** 1 Department of Botany, School of Basic and Applied Sciences, Central University of Punjab, Bathinda, Punjab 151401, India; alkaraniraj@gmail.com (A.R.); ksaini523@gmail.com (K.C.S.); felix.bast@gmail.com (F.B.); 2 Department of Chemical Engineering, Materials and Environment, Sapienza University of Rome, 00184 Rome, Italy; roberto.lavecchia@uniroma1.it; 3 Department of Biological Engineering, College of Engineering, Konkuk University, Seoul 05029, Korea; shashibiotechhpu@gmail.com

**Keywords:** oxidative stress, natural antioxidant, astaxanthin, mycothiol, peroxiredoxin, microalgae

## Abstract

Oxidative stress originates from an elevated intracellular level of free oxygen radicals that cause lipid peroxidation, protein denaturation, DNA hydroxylation, and apoptosis, ultimately impairing cell viability. Antioxidants scavenge free radicals and reduce oxidative stress, which further helps to prevent cellular damage. Medicinal plants, fruits, and spices are the primary sources of antioxidants from time immemorial. In contrast to plants, microorganisms can be used as a source of antioxidants with the advantage of fast growth under controlled conditions. Further, microbe-based antioxidants are nontoxic, noncarcinogenic, and biodegradable as compared to synthetic antioxidants. The present review aims to summarize the current state of the research on the antioxidant activity of microorganisms including actinomycetes, bacteria, fungi, protozoa, microalgae, and yeast, which produce a variety of antioxidant compounds, i.e., carotenoids, polyphenols, vitamins, and sterol, etc. Special emphasis is given to the mechanisms and signaling pathways followed by antioxidants to scavenge Reactive Oxygen Species (ROS), especially for those antioxidant compounds that have been scarcely investigated so far.

## 1. Introduction

Microorganisms are a diverse group of microscopic organisms including archaea, bacteria, fungi, protozoa, algae, and viruses. Microbial diversity produces a massive pool of unique chemicals, which have become a valuable source for innovative biotechnology. About 23,000 secondary metabolites from microorganisms are known, out of which actinomycetes exclusively produce approximately 42%, whereas fungi form almost similar amounts (42%), and the remaining 16% is produced by eubacteria [1]. Microbial secondary metabolites, including growth hormones, pigments, antibiotics, antitumor agents, etc., are not utilized for their growth and development, yet they have been revealed to have an excellent perspective for human health. These bioactive compounds have become significant sources of life-saving drugs [2]. These secondary metabolites are chiefly produced due to the induction of some cryptic gene clusters, which are generally inactive under normal conditions; hence, their expression would be significant in exploiting microorganisms’ chemical diversity. Many of these secondary metabolites hold specific antioxidant potential. Such bioactive molecules act as a valuable components for biotechnological applications, specifically for pharmaceuticals, nutraceuticals, and cosmetic industries.

This review comprehensively reports on the most important bioactive compounds found in actinomycetes, bacteria, archaea, fungi, microalgae, yeast, and protozoa with a special focus on their antioxidant potential. According to the Business Communication Company (BCC), in 2014, the total worldwide market for microbial products was estimated to be approximately $143.5 billion. It was expected to increase up to $306 billion from 2015 to 2020, at a compound annual growth rate (CAGR) of nearly 14.6%. Advancements in new technologies and economic advantages are substituting synthetic products and production processes with microbial products. The primary end-user market for microbes and microbial products is the healthcare sector, which was about $100.4 billion in 2014, approximately $111.5 billion in 2015, and was expected to increase to $187.8 billion in 2020 [3,4].

Microorganisms produce various bioactive compounds with antioxidant properties. Sen et al. [5] highlighted the classification of microbial antioxidants based on their chemical structure. They discussed the challenges faced during the development of food-grade microbial pigments and the use of cost-effective and advanced techniques to increase the production and shelf life of microbial pigments, eventually lowering the production costs. Chandra et al. [6] explored and summarized the antioxidant potential of microorganisms and throw light on using these bioactive metabolites for food, agriculture, pharmaceutical, and nutraceutical industries. The present review proceeds with the actinomycetes, exploring the potent antioxidant compound mycothiol, its metabolism, and mechanism to detoxify electrophiles, arsenate, H_2_O_2,_ etc., and also explores other antioxidants reported from them. The later section includes the antioxidants reported from archaea and bacteria. Mishra et al. [7] briefly reviewed some probiotic strains’ antioxidant properties, but they failed to dig into the pathways and signaling mechanisms followed by them to suppress oxidative stress. In comparison, Feng et al. [8] reviewed the antioxidant capacity, mechanisms, and signaling pathways followed by probiotic bacteria to overcome oxidative damage. They also discussed the genes associated with lactic acid bacteria (LAB) redox potential and suitable methods to evaluate LAB’s antioxidant capacity, yet the current research scenario’s information is lacking.

A detailed investigation of microalgal antioxidant, astaxanthin, increasing demand in the last few decades, and mechanism to detoxify ROS is elaborated in this review. Young and Lowe [9] assessed the antioxidant activities of carotenoids from plants and algae. Ekpe et al. [10] reviewed the antioxidant activities of astaxanthin and how it protects against various diseases. Davinelli et al. [11] evaluated the photoprotective, antioxidant, and anti-inflammatory effects of astaxanthin in skin physiology. However, these studies are unable to explore the detailed mode of action of astaxanthin to inhibit oxidative stress. 

Although fungi are a vast group comprising of 3.8 million species, yet very few reports are available on antioxidant compounds produced by them. Khatua et al. [12] explored the antioxidants potential of mushrooms, whereas Gupta et al. [13] reviewed the antioxidant compounds from fungal endophytes and recently Vitale et al. [14] revised the antioxidant molecules from marine fungi focusing on the production methodologies and their perspectives. The present review explored the latest antioxidant compounds discovered from endophytic fungi and mushrooms. 

Protozoa and yeast are the least explored; not much detail is available on their antioxidant potential. Here, we discuss the antioxidant potentials of yeast and protozoa with a detailed description of peroxiredoxins (Prxs) from yeast. Though many studies are available on microorganisms’ antioxidant potential [6,8,15,16], there have been comparatively very few studies that illustrate the various antioxidant bioactive compounds from microbial sources and their mode of action to scavenge free radicals. We analyse the signaling pathways by which mycothiol, astaxanthin, LAB, and Prxs to cope up with oxidative stress and finally summarize the prospect and challenges to be overcome to find out novel and potent antioxidant microbial compounds.

## 2. ROS (Reactive Oxygen Species) and Antioxidants 

Life on earth cannot be possible without oxygen; however, it can be harmful to life by instigating oxidative stress in cells and tissues due to ROS formation. Several sources and mechanisms advocated have contributed to the generation of these ROS. Free radicals are highly unstable ions, atoms, or molecules carrying an unpaired electron derived from oxygen, nitrogen, and sulfur. Oxygen-centered ROS include superoxide (O_2_^−^), peroxyl (ROO·), hydroxyl (HO·), nitric oxide (NO), and alkoxyl (RO·). The hydroxyl and the alkoxyl free radicals with a half-life of 10^−9^ s are highly reactive and speedily attack the molecules in adjacent cells, causing metabolic malfunctioning, destruction to cellular proteins, nucleic acids (RNA and DNA), lipids, and ultimately cell death [17]. The damage instigated by ROS and RNS (reactive nitrogen species) is unescapable and is only dealt with repair processes occurring within a cell [18,19]. Along with these ROS radicals, living organisms possess some nonradical ROS, including the singlet oxygen (^1^O_2_), hypochlorous acid (HOCl), and hydrogen peroxide (H_2_O_2_). The devastating ROS effect occurs on mitochondria, which produces ATP via oxidative phosphorylation and the electron transport chain [20]. 

**Figure 1:**
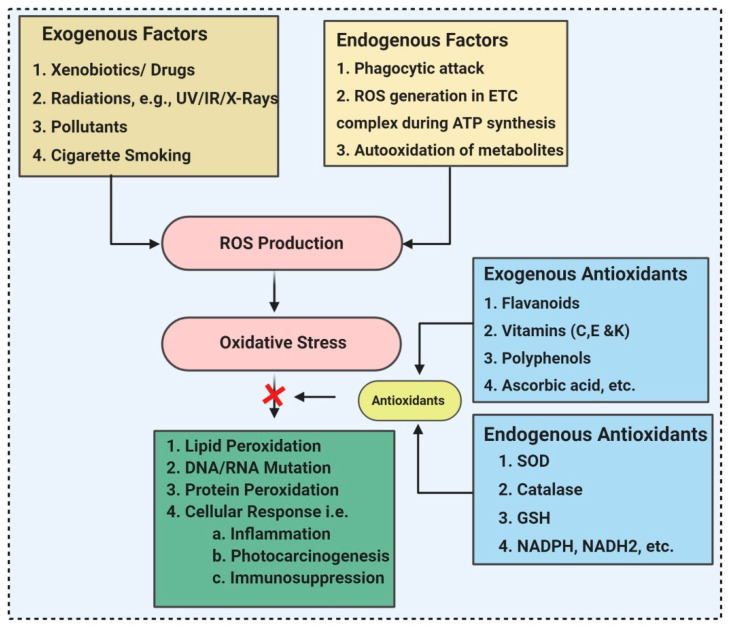
Various sources of oxidative stress and antioxidants.

Antioxidants are ROS scavengers that can shield, scavenge, and repair oxidative damage, thereby defending target assemblies or molecules from oxidative damages. According to their mode of action, antioxidants are either primary or secondary antioxidants. Primary antioxidants nullify free radicals by two mechanisms. One is by donating an H-atom known as hydrogen atom transfer (HAT), and the other is through a single electron transfer (SET) mechanism. These antioxidants are required in a lesser amount to neutralize a massive sum of free radicals. For example, phenolic antioxidants have high catalytic properties and can be easily regenerated [21]. The secondary antioxidants neutralize the ROS through prooxidant catalysts such as β-carotene, which neutralize ROS like singlet oxygen by quenching free radicals and are thus exhausted. Both primary antioxidants and secondary antioxidants can be either synthetic or natural [21]. Synthetic antioxidants, such as butylated hydroxytoluene (BHT), tertbutyl hydroquinone (TBHQ), butylated hydroxy anisole (BHA), and propyl gallate (PG) has been used to prevent lipid peroxidation (LPO) in food products. However, these synthetic antioxidants are cheap and stable at extreme ranges of environmental conditions, but they negatively impact the health, causing toxicity that endorses DNA damage (Figure 1). Dietary antioxidant reduces the prevalence of disorders including inflammation, cancer, aging, cardiovascular disease, diabetes, nephrotoxicity, cataract, and neurodegenerative disorders. Dietary antioxidants are previously reported from plants [22,23] and spices [24], but microorganisms remain the least explored in the case of antioxidants. Since the early 1980s, antioxidants from microorganisms were identified and used in healthcare due to their valuable therapeutic efficacy and accessibility [25]. Natural microbial biomolecules with antioxidant activity are described in Table 1. Antioxidants protect against ROS and enhance human physiological functions, consequently assisting in sustaining a healthy state and defending against ailments. Living organisms have enzymatic and nonenzymatic antioxidant mechanisms for inactivating ROS. Enzymes, including catalase (CAT), glutathione peroxidase, and superoxide dismutase, are the endogenous antioxidants that control ROS damage, whereas carotenes, flavonoids, reduced glutathione, bilirubin, coenzyme Q, and vitamin C are the sources of exogenous antioxidants.

After exposure to a maximum ROS concentration, sometimes the endogenous antioxidant system is conceded and is not fully functional. Therefore, to reimburse scarcity of antioxidants, exogenous antioxidants are supplied through food or nutritional supplements. Antioxidants play a crucial role in inhibiting oxidative damages and aid in preventing diseases like degenerative neuropathies, cardiovascular diseases, and cancer, as well as exerting anti-inflammatory, antiviral, and antiaging activities. 

Many microorganisms, including both Gram- and Gram+ bacteria, act as a natural source of antioxidants. The short hydrophobic peptide (Ser–Ser–Gln) fraction extracted from marine bacteria, *Kocuria marina* CDMP 10, from the Gulf of Mannar, India, is demonstrated as a potent free radical scavenger in hepatocellular carcinoma cell lines, signifying it as a potent pharmaceutical candidate with antioxidant activity [26]. Widowati et al. [27] evaluated the antioxidant potential of methanol extracts from microalgae, *Dunaliella salina, Tetraselmis chuii,* and *Isochrysis galbana* clone Tahiti, using 2,2-diphenyl-2-picrylhydrazyl (DPPH) assay. *I. galbana* clone Tahiti with a total phenol content of 17.798 mg GAE g-1 (Gallic Acid Equivalent), showed the highest antioxidant activity with 61.64% of inhibition at 50 ppm, followed by *D. salina* with 58.45% and *T. chuii* with 52.58% of inhibition. A study by Wali et al. [28] showed that *Nannochloropsis oculata*, contains a higher amount of polyphenol and exhibited in-vitro ROS scavenging activity, with an IC50 of 52.10 ± 0.85 µg/L.

## 3. Microorganisms as a Source of Antioxidant Compounds

### 3.1. Actinomycetes

The actinomycetes are gram-positive, aerobic, filamentous, and spore-forming bacteria, with a superior reputation in producing different kinds of metabolites with a broad spectrum of biological activities, including antioxidant, antifungal, antibacterial, and insecticidal activities. 

Mycothiol (MSH), a principal “sugar” thiol present in the cell wall of actinomycetes, serves as the glutathione (GSH) analogue. Actinomycetes lack the enzymes for GSH biosynthesis, but as a substitute, they utilize alternative low molecular weight thiols (LMW), such as bacillithiol (BSH) and MSH, ergothioneine (ESH) [56]. MSH maintains the intracellular redox homeostasis, allowing the appropriate working of many biological processes, counting enzyme activation, DNA synthesis, and cell-cycle regulation. MSH is a potent antioxidant, which acts as an electron donor/acceptor and assists as a cofactor in detoxifying free radicals, xenobiotics, and alkylating agents. Unlike GSH, MSH has two sugar components viz. *N*-glucosamine, inositol, and cysteine components as an alternative to the two amino acids, glycine, and glutamic acid [56]. 

#### 3.1.1. Biosynthesis of MSH

**Figure 2:**
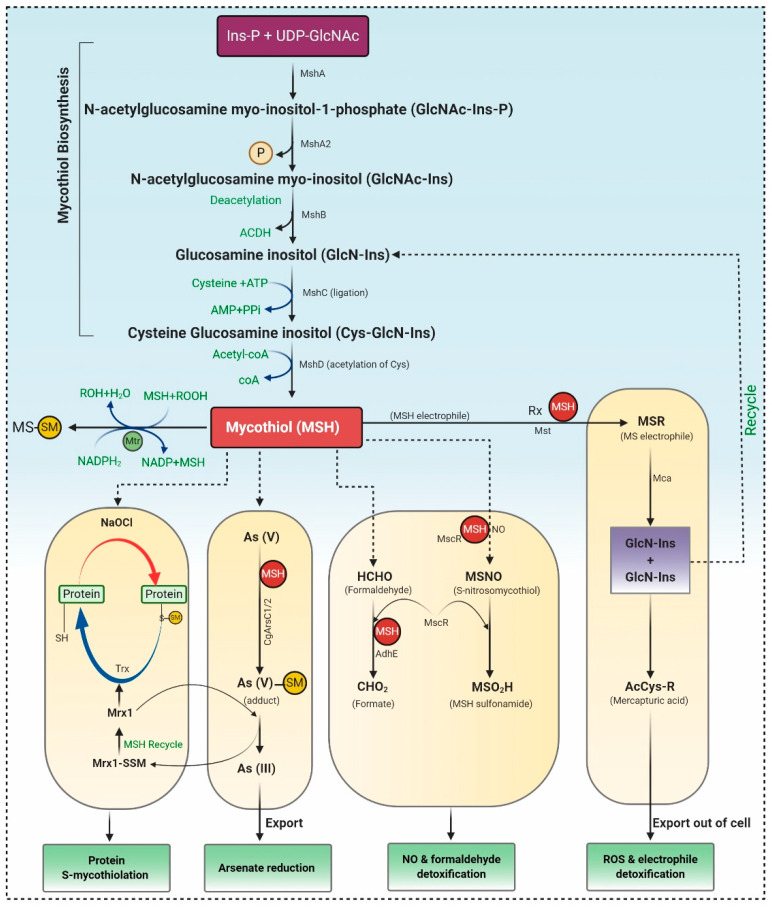
MSH biosynthesis and regulation in actinomycetes. Synthesis of MSH is catalyzed by five enzymes, including MshA, MshA2, MshB, MshC, and MshD. 1) Under Reactive Oxygen Species (ROS), MSH is oxidized to MSSM, which is further reduced by Mtr. 2) *S*-mycothiolation and protein regeneration occur via Mrx1 (mycoredoxin 1) /MSH/Mtr and thioredoxin/Thioredoxin reductase (Trx /TrxR) pathway. 3) Arsenate reductases CgArsC1/CgArsC2 along with MSH and Mrx1 reduced As (V) to As (III), which is exported through ABC transporter. 4) MSH acts as thiol cofactor for alcohol dehydrogenase MscR and formaldehyde dehydrogenase AdhE and is involved in NO and formaldehyde detoxification. 5) Mycothiol amidase (Mca) and mycothiol-S-transferases are involved in ROS and xenobiotic detoxification.

MSH biosynthesis involves five enzymes, and the substrates *myo*-inositol-1-phosphate (Ins-P), UDP-*N*-acetyl glucosamine (UDP-GlcNAc), and Cysteine (Cys) represented in Figure 2. Firstly Ins-P is conjugated with UDP-GlcNAc to yield *N*-acetyl glucosamine *myo*-inositol-1-phosphate (GlcNAc-Ins-P) via enzyme glycosyltransferase MshA. The MSH phosphatase MshA2 dephosphorylated GlcNAc-Ins-P, which is further deacetylated by the metal-dependent deacetylase MshB generating glucosamine inositol [1-*O*-(2-amino-1-deoxy-a-Dglucopyranosyl)-d-myo-inositol]. The Cys ligase MshC inserted Cys to yield Cys-GlcN-Ins. Finally, the Cys amino group is acetylated by the MSH acetyltransferase MshD to produce MSH [57].

#### 3.1.2. The Catalytic Action of the Mycothiol Disulfide Reductase (Mtr)

During the oxidative stress, MSH is oxidized to mycothiol disulfide (MSSM), which is further reduced by the NADPH-dependent Mtr. Mtr is the key enzyme involved in retaining MSH levels, which reduces the oxidized MSH disulfide. Mtr is a homodimeric flavoprotein disulfide isomerase that requires a cofactor, viz. FAD (flavin adenine dinucleotide). The oxidized Mtr (Mtrox) comprises a disulfide bridge between Cys39 and Cys44, which further reduces by accepting electrons from NADPH through FAD cofactor to produce reduced Mtr, as depicted in Figure 2. MSSM is confronted via the exchange of Cys39, resulting in the generation of Cys39-SSM, i.e., reduced MSSM and discharge of the first MSH moiety. Consequently, the disulfide bond in Cys39-SSM is condemned by the thiolate present at Cys44 forming Mtrox [58,59].

#### 3.1.3. Mycothiol-Dependent Detoxification of Electrophiles

MSH *S*-transferases (Mst) conjugate with MSH electrophiles (RX), creating MS-electrophiles (MSR), MSH *S*-conjugate amidase (Mca) cleave an amide bond of MSR to form a glucosaminyl inositols (GlcN-Ins) and mercapturic acid (AcCys-R). AcCys-R or MSH-S-associates of antibiotics or toxins are expelled from cells via ABC transporters, whereas GlcN-Ins is salvaged back to mycothiol (Figure 2) [58,59].

#### 3.1.4. Detoxification of Nitric Oxide (NO)

Detoxification of NO requires MSH-dependent enzyme nitrosothiol reductase (MscR) that exhibits S-nitrosomycothiol (MSNO) reductase activity, thereby generating MSH sulfonamide (MSO_2_H). Both MSH-dependent formaldehyde dehydrogenase AdhE and MscR oxidize formaldehyde to formate. In *Corynebacterium’s glutamicum*, maleylpyruvate is a ring fission output of gentisic acid and is converted to fumarylpyruvate through gentisate pathway by the MSH-dependent maleylpyruvate isomerase (Figure 2) [59,60].

#### 3.1.5. Detoxification of Arsenate

Arsenate detoxification is catalyzed by the arsenate reductases (CgArsC1/CgArsC_2_), which associate arsenate As (V) to MSH, generating As (V)-SM adduct, that is later on reduced by mycoredoxin-1 (Mrx1), forming Mrx1-SSM intermediate and As (III). As (III) is disseminated from the cells using two arsenite permeases of the Acr3 family. However, Mrx1-SSM depends on MSH for the regenerating Mrx1 [58,61].

#### 3.1.6. Protein *S*-Mycothiolation under NaClO and H_2_O_2_ Stress

Proteins are *S*-mycothiolated and recreated by the Mrx1/MSH/Mtr and Trx/TrxR pathways during NaOCl and H_2_O_2_ stress. These proteins control the activity of mycothiol peroxidase (Mpx), thioredoxin peroxidase (Tpx), and methionine sulfoxide reductase A (MsrA) in vitro [58]. Mpx and MsrA result in the formation of intramolecular disulfides and *S*-mycothiolations and involve both the Trx and Mrx1 pathways for reformation [62]. The Mrx1/Mtr/MSH pathways are likewise involved in reducing the peroxiredoxin AhpE in *Mycobacterium tuberculosis* (Figure 2). Therefore, it can be concluded that mycothiol is a promising and suitable candidate for antioxidant therapeutics. Only a few studies investigated the antioxidant compounds from actinomycetes. Yang et al. [42] reported the production of 1”-O-methyl-8-hydroxymethyl-daidzein, an isoflavone from *Streptomyces* sp. YIM 65408, with ROS scavenging activity. Zhou et al. [63] reported antioxidant, 2,6-dimethoxy terephthalic acid (IC_50_ 4.61mg/mL) from endophytic *Streptomyces* sp. YIM66017.

Chandra et al. [6] extensively reviewed the availability of different reactive functional groups responsible for the antioxidant activity of terpenoids, isoflavonoids, 4’, 7, 8-Trihydroxyisoflavone, naphterpins B and C, carazostatin, and carbazomycin B, isolated from *Streptomyces* sp. The first glycosylated phenazine derivative, phenazoviridin reported from *Streptomyces* sp., acts as a strong inhibitor of lipid peroxidation. Phenazoviridin has higher defending activity against KCN-induced acute hypoxia in mice than indeloxazine [64]. Thiazostatins A and B and benzastatins A to D produced by *S. tolurosus* and *S. nitrosporeus* were nitrogen-holding antioxidants, which either bears a tetrahydro quinolone ring or *p*-aminobenzamide unit. Benzastatins molecule comprises of N-H and one hydroxyl group, while benzastatins derivatives have methoxy group. Due to these functional groups’ presence, weaker lipid peroxidation compared to vitamin E was demonstrated from these compounds [65,66]. Several compounds such as antiostatins A1 to A4 and B1 to B4 reported from *S. cyaneus*, and carbazoquinocins A to F isolated from *S. violaceus*, were composed of carbazole comprising an o-quinone. Neocarazostatins A to C purified from the mycelium of *Streptomyces* sp. confirmed antioxidant activity [67]. Therefore, carbazole compounds produced by *Streptomyces* sp. contributed to be the major class of antioxidants. Tan et al. [68,69] demonstrated antioxidant activity of *Streptomyces* sp. MUM212 and *Streptomyces* sp. MUM265, concluded that both strains contain phenolic compounds that inhibit lipid peroxidation and reduce ROS due to their hydrogen-donating and electron transferring capabilities. Praptiwi et al. [70] reported organofluorine, a potent antioxidant from *Streptomyces* sp. strain TC1. Therefore, it can be considered that microbes are a potential candidate for production of bioactive compounds.

### 3.2. Archaea

Archaea specifically grow in harsh environmental conditions such as significantly higher or lower temperature, pH, salinity, etc. Halophilic archaea are a more promising candidate for producing carotenoids, thereby they show red and orange colored colonies. A universal purpose of carotenoids is their antioxidant activity leading to the protection of cells against oxidative stress, thus benefiting human health. Most haloarchaea biosynthesized bacterioruberin (BR), a C50 carotenoid, and its precursor bis-anhydro bacterioruberin (BABR), 2-isopentenyl-3,4-dehydrorhodopin (IDR), and mono-anhydro bacterioruberin (MABR) are represented in Table 2 [71]. BR present in the cell membrane helps halo archaeal cells acclimatize to hypersaline environments, resulting in stabilizing the cell membrane under such stress. Rodrigo et al. [72] investigate and described the haloarcheal BR production and its applications in biomedicine. In contrast, Giani et al. [71] elaborated BR and its derivatives’ production at mid-and large-scale and discussed its recent biotechnology and biomedicine applications. 

BR acts as a ‘‘rivet” and affects membrane fluidity by imitating as a water barricade and permitting permeability to oxygen and other molecules [73]. In BR, 13 pairs of conjugated double bonds are present compared to the nine pairs of conjugated double bonds present in β-carotene. This transformation makes BR a better ROS scavenger as compared to β -carotene [74]. BR offers resistance to γ irradiation, intense light, and DNA damage caused by UV irradiation, radiography, and H_2_O_2_ exposure [18]. Carotenoids from *Halorubrum* sp. BS2 reported having extraordinary antioxidant capacity compared to ascorbic acid [75]. The antioxidant capabilities of carotenoids produced by *Haloterrigena turkmenica, Haloferax volcanii, Halococcus morrhuae, Halogranum rubrum,* and *Halobacterium salinarum* were significantly higher than β-carotene [71]. Hyperthermophilic archaea are furnished with a wide range of antioxidant enzymes that play a crucial role in protecting the living cells from oxidative damage. These enzymes, along with protein disulfide oxidoreductase, thioredoxin, and thioredoxin reductase, act as a core of the antioxidant system and maintain redox homeostasis. As demonstrated by recently published data, the mainstream of aerobic hyperthermophilic archaea utilizes peroxiredoxins (Prxs) and thiol-dependent peroxidases to scavenge peroxides. LMW thiols act as cofactors in the detoxification of xenobiotic compounds. Newton and Javor [76] reported γ-glutamylcysteine (γGC), the first LMW thiol isolated from halophilic archaeon (haloarchaeon) *Halobacterium salinarum*. Later on, it was reported from other haloarchaea, including *Halobacterium saccharovorum*, *Haloarcula* (*Halobacterium*) *marismortui*, *Haloarcula californiae*, *Halococcus* sp. LS-1), and *Haloferax* (*Halobacterium*) *volcanii* [77,78]. Bacterioferritin comigratory protein 1 (Bcp1) belongs to the Prx family, isolated from hyperthermophilic archaeon *Sulfolobus solfataricus*, which has been characterized as an antioxidant that inhibits H_2_O_2_-induced apoptosis in H9c2 rat cardiomyoblast cells [79]. A 22-kDa protein, structurally similar to a class of DNA-binding protein (Dps) was isolated from hyperthermophilic acidophile *Sulfolobus solfataricus*, demonstrated to protect nucleic acids by defending DNA from oxidative damage and eliminating constituents that contribute to the formation of hydroxyl radicals [80].

### 3.3. Bacteria

Bacteria are closely associated with all life forms on earth and possess the ability to produce ample extracellular metabolites that also have antioxidant activities as represented in Table 1. In bacteria, carotenoids are produced by the extremophiles, including *Thermus filiformis*, *Micrococcus freudenreichii*, *Flavobacterium* sp., *Serratia marcescens*, *Agrobacterium* sp., *Pseudomonas aeruginosa*, *Rheinheimera* sp., and *Chromobacterium* sp. *Thermophilic* bacterium produces all-trans-zeaxanthin, zeaxanthin monoglucoside, thermobiszeaxanthins, and thermozeaxanthins [69]. Carotenoid ingestion might decrease the threat of diseases linked with oxidative stress; therefore, it acts as a proficient scavenger of ROS, RNS, singlet oxygen species (^1^O_2_), and nonbiological radicals [81]. 

Correa et al. [82] analyzed that when Antarctica bacteria belonging to the *Pedobacter* genus were exposed to cold temperatures and high UV radiation, they had developed an important antioxidant system and produce a variety of pigments that belongs to the carotenoids group and are capable of preventing oxidative damage. The antioxidant capacity of a mix of pigments viz. yelcho2, β-Carotene, and α-Tocopherol, was analyzed using three different methods viz. DPPH, ROS detection, and an oxygen electrode. In December 1988, the Marine Biotechnology Institute Co., Ltd. (MBI, Kamaishi, Japan) was established and began the isolation of novel or rare marine bacteria; many among them have been revealed to yield dicyclic or monocyclic C40 carotenoids, along with several acyclic C30 carotenoids [66]. MBI reported that *Paracoccus* sp. strain N81106 [83], *Brevundimonas* sp. strain SD212 [84], and *Flavobacterium* sp. PC-6 [85] produces astaxanthin glucoside, 2-hydroxyastaxanthin and 4-ketonostoxanthin 3′-sulfate, respectively. These are novel dicyclic C40 carotenoids with β-carotene (β, β-carotene) skeleton. The carotenoid biosynthesis gene cluster responsible for astaxanthin manufacturing was reported from the marine bacterium *Agrobacterium aurantiacum* [63]. This carotenoid gene cluster consists of five carotenogenic genes viz. *crtB, crtW, crtZ, crtY*, and *crtI* with the same orientation. Mishra et al. [86] investigated the capability of a semiquinone glucoside derivative (SQGD) from a *Bacillus* sp. INM-1 towards SOD, catalase, GSH, GST antioxidant enzymes. There was a significant increase in SOD (35%) activity and GST level (0.46 ± 0.03 μmol/min/mg of protein) in mice’s kidney after SQGD treatment compared to untreated control mice within 12–72 h. Sy et al. [87] demonstrated that the GI (gastrointestinal) tract sometimes undergoes considerable oxidative stress in postprandial circumstances when dietary iron is exceedingly existing in food both as heme or free form [87]. Excessive iron accumulation results in lipid peroxidation, specifically in acidic conditions. Sy et al. [87] isolated *Bacillus indicus* HU36, producing carotenoids from human fecal and assessed the stability (sensitivity to iron-induced autoxidation) and antioxidant activity (inhibition of iron-induced lipid peroxidation). Photolo et al. [88] reported *Methylobacterium radiotolerans* MAMP 4754 (IC_50_ of 5.65 μg/mL) contains 9-octadecene, 2,4-dinitrophenyl acetate, and 2(5H)-furanone that can scavenge ROS [88]. Violacein, a purple pigment is a potent antioxidant primarily produced by *Chromobacter violaceum* and *Pseudoalteromonas*, stimulating mucosal defense mechanisms for protection against oxidative damage due to gastric ulcers [34]. Staphyloxanthin, a yellow pigment produced by *Staphylococcus aureus*, prevents carbon tetrachloride-induced oxidative stress in Swiss albino mice [89].

Several studies focused on the antioxidant activities of the bacterial extracellular polysaccharides (EPS) [90,91,92,93,94], which are nontoxic polymer and extensively used in nutraceuticals, pharmaceuticals, cosmetics, and food industries. Shindo and Misawa [66] investigated the structures and antioxidant activities of newly reported acyclic carotenoids with a C30 diapolycopenedioc acid, aglycone xylosylesters A–C and methyl 5-glucosyl-5,6-dihydro-apo-4,4′-lycopenoate, and monocyclic C40 carotenoids, (3R,2′S)-myxol, and (3R)-saproxanthin, but they did not trace their metabolic and signaling pathways. Lin et al. [93] elucidated the antioxidant potential and described the sugar composition of DeinoPol, a deinococcal EPS. Wang et al. [94] thoroughly summarize the in vitro studies and investigated the up-to-date advancements and discoveries considering bacterial polysaccharides as a reducing agent, ROS scavenger, and metal chelator. However, it did not cover the in vivo studies needed to pass the clinical trials for their acceptance as a novel antioxidant. Extensive studies should be conducted in the future focusing on both in vitro and in vivo antioxidant activities of polysaccharides, and experiments should be designed to extract high-purity polysaccharides from microbial sources using cost-effective technologies. 

**Figure 3:**
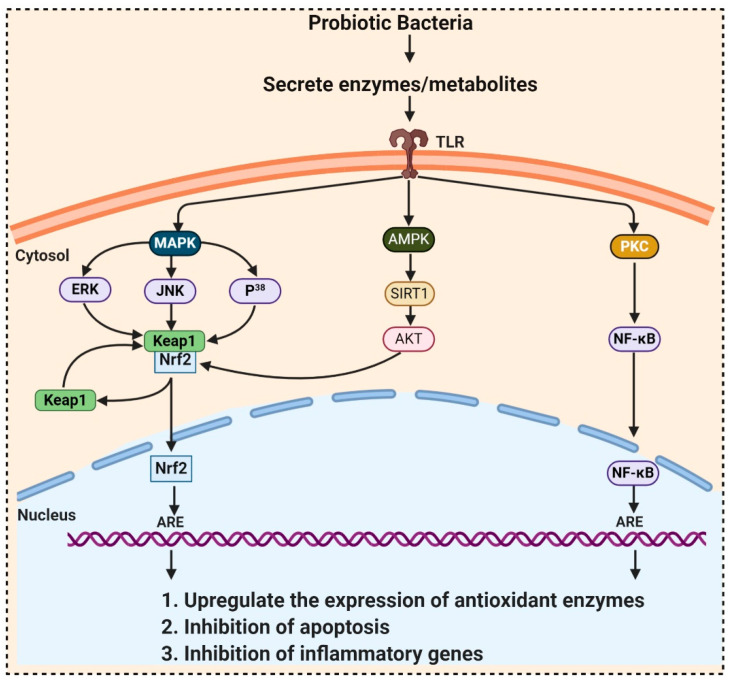
Probiotic activates metabolites and enzymes that enter the cell through toll-like receptors (TLR) and induced Nrf2-Keap1-ARE pathway. During ROS attack, the redox-sensitive cysteine residues of Keap1 react and disrupt the functional conformation of Keap1, thereby activating Nrf2. Nrf2 translocates towards the nucleus and binds to antioxidant response element (ARE) sequences, activating the transcription of ARE-driven genes, encoding antioxidant enzymes, and detoxifying proteins. ROS mediates the expression of redox-sensitive transcription factor NFκB and further expression of inflammatory cytokines. ROS also activates SIRT1 (Silent information regulator T1), mediated Adenosine monophosphate (AMP)-activated protein kinase (AMPK) pathway.

Probiotics contain billions of bacteria that are capable of fermenting foods and beverages and are also used as a supplement due to their numerous beneficial activities including antioxidant properties. They accumulate in the GI tract and produce metabolites, which possess antioxidant activities. Probiotic bacteria have unique antioxidant enzyme systems that stimulate the host antioxidant system. Research on pigs exposed that dietary *Lactobacillus fermentum* elevated the serum SOD, glutathione peroxidase (GPx) and hepatic CAT, and Cu and Zn-SOD than the control group. Aarti et al. [95] reported the hydrogen peroxide resistant activity of *Lactobacillus brevis* LAP2, isolated from *Hentak,* a fermented fish food from Manipur, India. Son et al. [96], isolated *Lactobacillus plantarum* Ln4 and G72, from kimchi, exhibited (40.97%) DPPH, ROS scavenging, and β-carotene oxidation-inhibitory activities (38.42%). Another strain, SC61 of *Lactobacillus plantarum*, isolated by Son et al. [97] from jangajji, a Korean traditional fermented food, expresses a high level of IL-1β, IL-6, and TNF-α. These studies did not explore how the probiotic bacteria communicate with the signaling pathways, which is elaborated in Table 3 and Figure 3. In 2018, Ayyanna and colleagues assessed probiotic strains *Lactobacillus mucosae* AN1 and *Lactobacillus fermentum* SNR1 for a significant decrease in rat paw edema, induced by Freund’s adjuvant and carrageenan. This decrease in inflammation is due to the high expression of IL-10, an anti-inflammatory cytokine, inhibiting prostaglandins synthesis, thereby preventing autoimmune and inflammatory diseases [98].

Kullisaar et al. [112] reported that *Lactobacillus fermentum* E-3 and E-18 express Mn-SOD that resists oxidative stress. Probiotic bacteria’s ability to locally deliver SOD exposed a novel perspective to bowel diseases caused by ROS production [112]. LeBlanc and colleagues’ demonstrated that the engineered *Lactobacillus casei* BL23 produce SOD when given to mice with Crohn’s disease and mice showed a quicker recovery from weight loss, improved gut enzyme activities, and a lesser extent of intestinal inflammation than the control mice [113]. CAT also participates in cellular antioxidant defense by decomposing H_2_O_2_, but LAB is usually CAT-negative. LeBlanc et al. [114] evidenced that *Lactococcus slactis* produced CAT and prevented 1,2-dimethylhydrazine (DMH)-induced colon cancer in mice. It was also proved that engineered *Lactobacillus casei* BL23 produce CAT and decrease or prevent the brutality of intestinal pathologies instigated by ROS. An in vitro study by Wang et al. [106] reported that *Bacillus amyloliquefaciens* SC06 raised *CAT* and *GST* gene expressions and activity in intestinal porcine epithelial cells-1 (IPEC-1). It is still doubtful whether those in vitro and in vivo results are conveyable to humans because maximum probiotics cannot be colonized in the gut and are eliminated immediately after consumption.

### 3.4. Fungi

Whenever there is a discussion about fungal metabolites, the story of penicillin has been told many times. Alexander Fleming, in 1929 discovered that mold juice’ from *Penicillium notatum* had antibacterial action and named this biological activity “Penicillin” [88]. In the past few years, researchers instigated to look for a fungal strain that could be grown quickly and produce bioactive metabolites in submerged culture. Later on, *Penicillium chrysogenum* was selected for large-scale production of *Penicillium* [98]. A wide range of primary and secondary metabolites including flavonoids, phenols, steroids, alkaloids, xanthones, etc. produced by the most diverse group of fungi, have antioxidant activities, and are highly exploited by the therapeutical, pharmacological, and medicinal sector. Maximum therapeutical and pharmaceutical investigations focused on the species belonging to Ascomycota and Basidiomycota [39], which include endophytic fungi, exhibiting higher antioxidant potential. A wide range of natural products including antioxidants, anticancerous, antiviral, anti-insecticidal, immune-suppressant, antimycobacterial, antimicrobial, and antimalarial, has been reported from endophytic fungi [97].

Recently, many studies have guided the discovery of significant plant secondary metabolites from endophytic fungi, thereby confirming the viewpoint that endophytic fungi can act as a substitute for plant metabolites. From 1987 to 2000, around 140 new natural products were extracted from endophytic fungi [115]. Five percent of fungal species had been characterized to produce bioactive metabolites [96].

The discovery of antioxidant compounds, pestacin and isopestacin from *Pestalotiopsis microspore,* has opened a new direction towards investigating the antioxidant potential of endophytic fungi [95,116]. Zeng et al. [117] explored 49 endophytic fungi belonging to the family Xylariaceae, for in-vitro antioxidant activities. Among them, endophytic fungi from *Scapania verrucosa* were reported as a budding and novel source of natural antioxidants. Prihantini et al. [118] isolated seven fungal strains of the genus *Pestalotiopsis* from the leaves and stems of *Elaeocarpus sylvestris*. *Pestalotiopsis* sp. EST 02 and *Pseudocercospora* sp. ESL 02 possesses terreic acid and 6-methylsalicylic acid, having antioxidant activity. Nuraini et al. [119] reported that *Aspergillus minisclerotigens* AKF1 and *Aspergillus oryzae* exhibited antioxidant ability with an IC50 142.96μg/mL and 145.01μg/mL, respectively. GC-MS analysis confirmed that the most active compound isolated from AKF1 and DK7 was dihydropyran and 4H-Pyran-4-one,5-hydroxy-2-(hydroxymethyl-(CAS) Kojic acid, respectively. 

Exopolysaccharides and flavonoids produced by *Fusarium oxysporum* isolated by Caicedo et al. [120] also exhibit such properties. Methanolic extracts from *Monascus purpureus*, OYRM1, *P*. 32783, *P*. *salmoneo*-*stramineus,* and *T. versicolor* inhibit linoleic acid oxidation by at least 53% [121]. Total 42 fungal endophyte strains related to a medicinal plant, *Nerium oleander L.* (Apocynaceae) were isolated and screened for antioxidant and antimicrobial capacity. Out of these 42 isolated only *Chaetomium sp.* inhibit xanthine oxidase activity with an IC50 of 109.8 lg/mL, had an antioxidant activity with the highest level of phenolics [122].

#### 3.4.1. Mushrooms

In the last few decades, edible mushrooms have gained attention and are commercialized as a natural antioxidant source. Porcini (*Boletus edulis*) is one of the most common palatable mycorrhizal mushrooms in China also called “white bolete” in Yunnan, contains ergothioneine and glutathione, compounds with antioxidant activity, protecting mitochondrial components from oxidative stress [41,123]. Chen et al. [124] reported ergothioneine (ET) from the fruiting bodies of palatable species, *Pleurotus ostreatus*, *P. citrinopileatus*, and *P. salmoneostramineus*. ET plays a part in guarding mitochondrial apparatuses against oxidative damage caused by the mitochondrial generation of ^•^O_2_^−^. Edible mushrooms are a substantial source of vitamins (B_1_, B_2_, B_12_, C, D, and E), polyunsaturated fatty acids (PUFA), etc and are used as a vital source of home remedies against several ailments stimulated by oxidative stress in Asia [113]. The investigation of the methanolic extract of *Cantharellus cibarius* by Kozarski et al. [125] exhibited that phenols were major antioxidant components in them, followed by flavonoids. Polysaccharides ((1→6)-β-d-glucans) obtained from *Aspergillus brasiliensis* after pronase deproteinization had maximum antioxidant activity against ^•^OH and ^•^O_2_^−^ radicals. β-glycan is a major contributor to mushrooms’ antioxidant effects, which can be directly taken up by M cells of Peyer’s patches or dendritic cells activating systemic response in organisms [114,126]. 

Hispidin, a polyphenol isolated from mushrooms of the genera *Phellinus* and *Inonotus*, used as traditional Chinese medicine for hundreds of years, possesses antioxidant activities. Hispidin derivatives namely methylinoscavin A, inoscavin B, and methylinoscavin B were isolated from the fruiting bodies of *Inonotus xeranticus*. Methylinoscavin A (IC50 20 µM) and methylinoscavin B (IC50 38 µM) with methyl group exhibit lesser ROS scavenging than inoscavin B (IC50) 95 µM), without methyl group [127]. In 2011, Zan and his colleagues isolated inonotusin A and B, natural antioxidants along with hispidin, (E)-4-(3,4-dihydroxyphenyl) but-3-en-2-one, and 3,4-dihydroxybenzaldehyde, from the methanolic extract of the fruit bodies of *Inonotus hispidus* [128]. Li et al. [129] extracted hispidin from *Phellinus igniarius* and were reported to scavenge free radical, inhibit erythrocyte hemolysis, and lipid peroxidation.

Investigations mentioning the potential antioxidant activities of edible mushrooms are uncountable. Guo et al. [130] reported two new illudane sesquiterpenoids, one new menthane monoterpene, craterellins D and E, 4-hydroxy-4-isopropenyl-cyclohexanemethanol acetate, from *Craterellus cornucopioides* and also assessed its cytotoxic activities. Yang et al. [131] extracted and explored the antioxidant properties of a novel polysaccharide fraction from *C. cornucopioides*. Ao et al. [132] described antioxidant activity (IC50 47.5 μg/mL) of *Lentinus tigrinus* from Nagaland, India. Phenolic extract of *Agaricus* *brasiliensis* exhibited antioxidant activity, assessed by DPPH (50.64 μmolTE/g) and ABTS (128.60 μmolTE/g) assays [133]. ROS Scavenging property of the peel, gills, inner cap, and stipes tissues of *Agaricus bisporus* and *Cyclocybe cylindracea* (EC_50_ 58–89 μg/mL), was about 340 times higher than *Flammulina velutipes* inner cap (EC_50_ 19.570 mg/mL) [134]. Due to the higher percentage of antioxidant compounds, extract from these edible mushrooms can be applied as a natural antioxidant in the food industry. The health-promoting outcome of mushrooms has fascinated many groups during the past few years, because of the presence of a wide range of secondary metabolites in their fruiting bodies. Approximately 270 species of mushrooms are now recognized as most likely therapeutic agents, used to safeguard human health [135].

#### 3.4.2. Yeast

Yeasts are mostly reported from raw materials, meat, fruits, and food products such as yogurt, sausage, and cheeses. Yeasts usually produce antimicrobial compounds that inhibit the growth of harmful bacteria or mold. Some classes of yeasts secrete toxins, thereby naming them killer yeasts [107]. *Saccharomyces cerevisiae* (Baker’s yeast)*,* is the most widely studied yeast involved in biotechnological practices because of its adequate fermentation capacity [123]. Prior studies exhibited that genus *Saccharomyces* possess probiotic properties. *S. cerevisiae* IFST 062013 isolated from fruits, produce enzymes including β-galactosidase, lipase, protease, phytase, and L-asparaginase that have robust ROS scavenging activity. Production of astaxanthin from red yeast *Phaffia rhodozyma* was also reported [123]. 

The yeast’s antioxidant property is due to the high content of polysaccharides, β-d-glucans, and α-d-mannans existing in the yeast cell wall. These two cell wall polysaccharides were isolated from the industrial strains *Saccharomyces cerevisiae* and *Candida utilis.* β-d-glucans and α-d-mannans exhibited a protective effect of water-soluble carboxymethylated yeast (1→3)-β-d-glucan (CMG) alongside lipid peroxidation in phosphatidylcholine liposomes, which act as a cell membrane model. CMG can also inhibit peroxidation persuaded by ultraviolet radiation A (UVA), which causes singlet oxygen production. The use of *Saccharomyces* β-glucans had already been approved by the European Food Safety Authority (EFSA). *Saccharomyces* β-glucans, denoted as yeast beta-glucans are used as a food ingredient [136]. 

Torularhodin, a carotenoid extracted from red yeast *Sporidiobolus pararoseus* possesses a more vital ability to scavenge peroxide free radicals than carotenes also more potent than α-tocopherol in preventing lipid peroxidation. 

**Figure 4:**
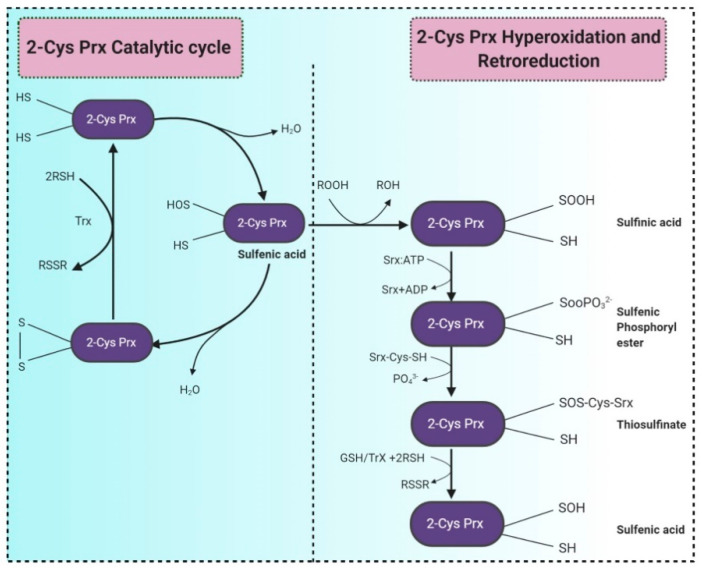
Two-step catalytic mechanism of 2-Cys Prx. The sulfhydryl group at the 2-Cys Prxs is oxidized to sulfenic acid (–SOH), which condenses with the –SH group at the cysteine from the other subunit forming an inter-subunit disulfide bond. This bond is further reduced by thioredoxin (Trx) or another reductase. Continuous peroxide signaling leads to reversible hyperoxidation and formation of sulfinic acid (–SOOH) at peroxidatic cysteine. Sulfinic 2-Cys Prx is reduced by sulfiredoxin (Srx) with an ATP’s consumption and generates a sulfinic phosphoryl ester intermediate. Cys 99 present in Srx reacts with this intermediate to form thiosulfinate that can be attacked by RSH to generate sulfenic acid. (RSH signifies a thiol equivalent such as glutathione, dithiothreitol, or thioredoxin). Adapted from [140].

Peroxiredoxins (Prxs) are discovered as an abundant proteins’ family of antioxidant enzymes, expressed ubiquitously, and act as peroxidases reducing H_2_O_2_ and alkyl hydroperoxides into water or alcohol, respectively. The Prx superfamily comprises two subgroups, 1-Cys Prx and 2-Cys Prx, based on one or two preserved cysteine residues (Figure 4) [102]. Prx has first discovered in *Saccharomyces cerevisiae* a protein that could prevent the reactivation of glutamine synthetase via the thiol/Fe (III)/oxygen oxidation system. The genome of *S. cerevisiae* encompasses a sequence (ORF YBL064C), which codes for a protein corresponding to human 1-Cys Prx [137]. Many studies reported two cytosolic thioredoxins, i.e., Trx1 and Trx2, from yeasts. Both are regenerated by cytosolic thioredoxin reductase Trr1, which utilizes NADPH as an electron donor. A study by Brachmann et al. (2020) reported several thiol-dependent oxidoreductases from *S. cerevisiae*, including glutathione peroxidases (Gpx1-Gpx3) and various peroxiredoxins (Ahp1, Dot5, Prx1, Tsa1, Tsa2) and depicted that Ahp1, detoxifies peroxides by bridging a disulfide bond between peroxidatic and resolving cysteines which further reduced by the thioredoxin system. Tsa1 is the major yeast peroxiredoxin, a moonlighting protein, also acts as a protein chaperone, a redox switch, specific antioxidant to protect the cell against the oxidative stress caused by nascent-protein misfolding and aggregation [138,139].

### 3.5. Microalgae

**Figure 5:**
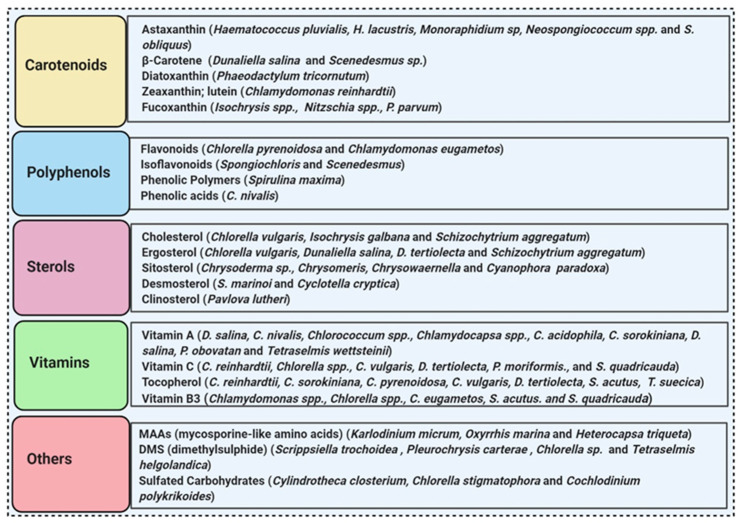
Some antioxidant compounds reported from microalgae.

More than 28,000 potential compounds are reported from microalgae, including hundreds of new compounds discovered every year [141]. Most of them have been described from *Chordata* (including ascidians) and *Porifera* (sponges), but they are hard to cultivate, and to attain a sustainable supply of the compounds of interest is problematic. In recent times, high interest is paid in analyzing microalgae’s biotechnological potential as they have short generation time, easier to cultivate, and signify an ecofriendly approach but a less explored resource for drug discovery. Microalgae are photosynthetic eukaryotes comprising one of the key members of freshwater and marine phytoplankton and are exceptional cradles of pigments, carotenoids, ω-3 fatty acids, lipids, sterols, and other biomolecules as depicted in Figure 5 [106]. More than 7000 species are enlisted as “Green microalgae” and they all grow in diverse habitats. Among them, *Haematococcus pluvialis* is the most important commercial microalgae and is also the richest source of natural astaxanthin which is regarded as “super anti-oxidant” [112]. Apart from *β*-carotene, astaxanthin has received considerable attention lately. Astaxanthin (3,3’-dihydroxy-β,β’-carotene-4,4’ -dione), a marine xanthophyll carotenoid was first isolated by Kuhn and Soerensen from a lobster [5]. Astaxanthin was initially used as a colorant for aquaculture, besides being approved as a coloring agent for food supplements since 1991. Due to emerging health benefits, demand for astaxanthin has increased rapidly in medicine, food industries, cosmetics, etc. In 2016 market share of astaxanthin was USD 512.8 million, which increased at a CAGR of 6.73% by 2017 and was expected to reach USD 814.1 million and USD 2.57 billion by 2022 and 2025, respectively, worldwide [30,142]. The same is the case with carotenoids, whose market demand was 1.24 billion USD (B$) in 2015 and is expected to rise to 1.6 B$ by 2023 with a CAGR of 3.5%. The demand for lutein in 2015 was 135 M$, which might escalate up to 200 M$ by 2024, with a CAGR of 5.3%. The hike in CAGR is due to the increasing demand for lutein-rich dietary supplements [143]. 

Polyene chain along with conjugated double bonds permit astaxanthin to pass through the lipid bilayer and allow the quenching of singlet oxygen and ROS removal [112,144]. 

**Figure 6:**
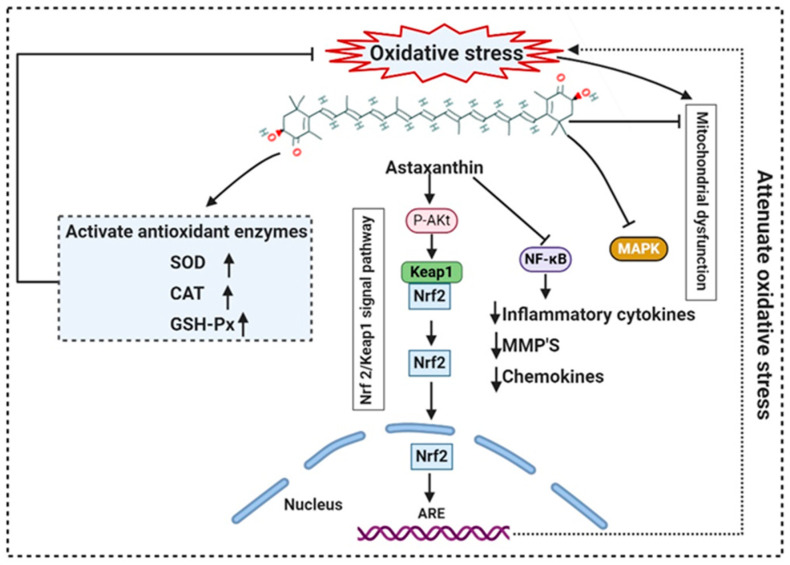
Mode of action of astaxanthin by inhibiting oxidative stress-induced mitochondrial dysfunction and activating transcription factor nuclear factor erythroid 2-related factor 2 (Nrf2). Astaxanthin suppresses nuclear expression of NF-κB and MAPK to reduce downstream production of proinflammatory cytokines. NF-κB: Nuclear factor-κB; MMP: matrix metallopeptidase; MAPK: mitogen-activated protein kinase; ARE: Antioxidant response elements.

The presence of hydroxyl and keto moieties on each ionone ring provides a unique structure to astaxanthin, permitting it to pass through the lipid bilayer membrane and allowing it to protect the cell membrane [112]. Unlike many antioxidants, which quench and scavenge ROS and other free radicals including, superoxide anion, hydrogen peroxide, singlet oxygen, etc.) either in the inner (e.g., vitamin E and *β*-carotene) or the outer side of the bilayer membrane (e.g., vitamin C), astaxanthin can act as both the inner and outer layers of the cellular membrane [145]. Many studies demonstrated that astaxanthin had 500 times higher antioxidant capacity than vitamin E, 65 times more potent than vitamin C, 54 times stronger than β-carotene, and ten times more potent than β-carotene, zeaxanthin, lutein, canthaxanthin. At present, approximately 95% of synthetic astaxanthin is available in the market, whereas only <1% natural astaxanthin derived from *H. pluvialis* is commercialized [146]. Astaxanthin upregulates the expression of Nrf2, whereas it simultaneously downregulates the Kelch-like ECH-associated protein-1 (Keap1) expression. In the presence of oxidative stress, Nrf2 dissociates from Keap1, translocates into the nucleus, binds to the ARE, and induces the expression of Phase II enzymes, as shown in Figure 6. Astaxanthin prevents lipid peroxidation by activating antioxidant enzymes involved in lipid metabolism, namely thioredoxin reductase (TrxR) and paraoxonase-1 [147,148,149]. Pongkan et al. [150] depicted higher levels of mtROS production, mitochondrial depolarization, and swelling in mitochondria isolated from the ischemic myocardium of mice astaxanthin treatment decreases mtROS production, mitochondria depolarization, and swelling.

Synthetic astaxanthin, synthesized through Wittig reaction between asta-C_15_ -triarylphosphonium salt and the C_10_-dialdehyde [151], but it has 20 times lesser antioxidant capacity as compared to its natural counterpart, and until today it has not been permitted for human consumption [16]. However, *H. pluvialis* has been approved as a color additive and dietary-supplement in the USA, Japan, and some European countries. Astaxanthin from *H. pluvialis* had been granted “GRAS” status (Generally Recognized As Safe) by US FDA (Food and Drug Administration) [152]. The European Food Safety Authority (EFSA) recommended the use of 0.034 mg/kg of the bodyweight of astaxanthin. Spiller and Dewell [153] reported that 2–4 mg of a daily dose of astaxanthin is safe whereas not toxicity was reported up to 6mg/day consumption. Recently, AIgatechnologies, Mera Pharmaceuticals Inc, Cyanotech Corporation, Fuji Chemical Industry Co. Ltd., etc. are involved in the commercial production of astaxanthin from *H. pluvialis*. Its global market is predicted to GRASP $2.57 billion by 2025 [30]. L-Ascorbic acid (vitamin C), present both in cytosol and chloroplast, reduce many ROS and act as a scavenger for hydroxyl radicals, superoxide, and lipid hydroperoxides. The maximum concentration of vitamin C was reported from *Chlorella* sp. [99] and *Dunaliella* sp. [100], *Skeletonema costatum,* and *Chaetoceros calcitrans*. Glutathione (GSH), a tripeptide (Glu-Cys-Gly), acts as a sequence breaker of ROS reactions and ascorbate regeneration. α-tocopherol is the most active antioxidant synthesized in the chloroplasts of *Dunaliella tertiolecta* and *Tetraselmis suecica*. The lipophilic carotenoids, synthesized in diatoms and dinoflagellates allow quenching of singlet oxygen [154], leading to the formation of a carotenoid triplet state (Equation (1)).

^1^O_2_ * + CAR → ^3^O_2_ + ^3^CAR* (1)

Lutein is a xanthophyll that defends long-chain polyunsaturated fatty acids. *Muriellopsis* sp. accumulate high lutein levels, whereas, in *Scenedesmus*, lutein and β-carotene production are accelerated by increasing both the pH and temperature throughout the cultivation [101]. The commercial source of lutein is *Muriellopsis* sp. 

β-carotene, isolated from the *Dunaliella salina*, is commercially produced for its antioxidant properties. Marennine is isolated from the marine diatom *Haslea ostrearia* also has antioxidant and antimicrobial activities [155]. Apart from diatoms, additional microalgae, including flagellates, dinoflagellates, and green algae, have also been scrutinized for potential biotechnological applications [156]. Carotenoid, fucoxanthin reported from brown seaweeds, and some diatom species have antioxidant, antidiabetic, antiangiogenic, anti-inflammatory, anticancer, and antimalarial activities. Methanolic extract of *Spirulina maxima,* and *Chlorococcum minutum* NIOF17/002 exhibit antioxidant activity with the IC_50_ of 0.18 mg/mL and 3.92 to 9.83 mg/mL of ascorbic acid, respectively [157,158]. 

### 3.6. Protozoa

Protozoa are regarded as the first and simplest organism of the animal kingdom discovered by Anton van Leeuwenhoek. Most protozoan species are unicellular, free-living, motile, and microscopic, whereas some species are mutualistic and parasitic. More than 10,000 protozoan species are described as parasitic [159]. *Entamoeba histolytica*, *Trypanosoma*, *Giardia*, *Leishmania*, *Trichomonads*, and *Plasmodium* are usually anaerobic but sometimes found to be microaerobic or microaerophilic and produce H_2_0_2_ as a product of metabolism. Some intracellular protozoans are manifest to the toxic oxygen metabolites produced by the host’s effector immune cells. Protozoa either lack or deficient in antioxidant enzyme systems including CAT, GSH, and SOD, necessary for the detoxification of H_2_O_2_. However, they have alternative detoxification mechanisms. A higher concentration of NADH-dependent oxidase and a lower concentration of NADH-dependent peroxidase activities were perceived to accomplish this task. *Entamoeba histolytica*, *Trichomonas vaginalis*, and *Tritrichomonas foetus* were reported to encompass NADH oxidase activity. Since protozoa are known to be parasitic, very few reports are available regarding their antioxidant activity. Mastronicola et al. [160] explored the antioxidant defense systems in *Giardia intestinalis*, which lacks conventional antioxidant enzymes; despite this, it can rely on a well-organized antioxidant system to survive the nitrosative and oxidative stress conditions. Joardar & Babu [161] reviewed the most recent work on parasitic thiol-based enzymatic antioxidant thioredoxin reductase (TrxR), and discuss how to treat filariasis, helminths, and other parasitic infections. Jeelani and Nozaki [162] reported that *Entamoeba histolytica*, an intestinal parasite, causes amebic dysentery, lacks eukaryotic antioxidative defense systems but owns an efficient thioredoxin system, composed of thioredoxin and thioredoxin reductase, both are crucial for antioxidant activity and sustaining cellular redox balance [162].

## 4. Future Prospectus

Oxidative stress occurs due to imbalance among reactive oxygen and nitrogen species (RONS) production and antioxidant defenses, which is a crucial factor, evolving various diseases such as cardiovascular diseases, chronic kidney disease, chronic obstructive pulmonary disease, neurodegenerative diseases by causing damage to the DNA, RNA, proteins, and other macromolecules, ultimately comprising human health. Antioxidants act as vital molecules that can degrade oxidants, exercise protection, and scavenge free radicals. Subsequently, the requirement for stable and promising antioxidants is rising globally, and it becomes crucial to explore natural antioxidants from various resources. Bioactive molecules producing microorganisms are relatively common in nature and are considered a significant source of therapeutic bioactive compounds that might advance the novel drugs. Microbial bioactive compounds include phenolics and flavonoids that exhibit a positive impact on health and well-being. Nowadays, about 60% of drugs available in the market are from natural sources. Microorganisms produce nearly 23,000 known secondary metabolites.

Nutraceuticals and the medical importance of microbial antioxidants are well documented, yet the research on antioxidant agents is a real and significant challenge of this century. Microorganisms are presently considered a significant source of therapeutic bioactive compounds that might lead to novel drug advancement. Apart from the high demand, the whole method of bringing a new antioxidant to the market is a long and complicated process for pharmaceuticals industries. It takes more than a decade to complete the entire course, with a maximum rate of unsuccessfulness. The antioxidants have to pass clinical trials before being approved by the national regulatory authorities for public sector use. Another major challenge is to elucidate the structural features of microbial antioxidants so that the addition and removal of essential elements and negative factors, respectively, can be proceeded to enable the discovery of novel antioxidants. Detailed structural knowledge would enable the understanding of antioxidant performance at both cellular and molecular levels. Thereby, future research must focus on the elucidation of antioxidant structure, physicochemical properties, and effects specifically on in vitro and in vivo models, enabling the disclosure of new strategies in current industrial sectors. 

## 5. Conclusions

The present review highlighted the potential of various microorganism to produce antioxidants and their importance as innovative sources of natural bioactive molecules. Microorganisms are easily cultivable and allow a production of natural antioxidants more efficient than in plants. Most microbial antioxidants are nonmutagenic and noncytotoxic compared to the synthetic ones. There is a vast diversity of microorganisms, but only few of them have been cultured and examined for secondary metabolite production. Microalgae, bacteria, yeast, actinomycetes, and mushrooms have revealed the presence of phenols, flavonoids, steroids, and alkaloids of great interest to the pharmaceutical and neutraceutical sectors.

Several molecules with promising antioxidant activity, such as astaxanthin, pestacin, isopestacin, and polysaccharides, are found in fungi and used as functional ingredients in food, nutraceutical, cosmetic, and pharmaceutical products. However, further investigations are required to improve the plethora of active metabolites already reported from fungi. In vitro and in vivo studies proved that ingestion of LAB decreased lipid peroxidation and enhanced the activity of CAT, SOD, and GPx, modulating the redox state of the host cells by regulating signaling pathways. Nevertheless, a better knowledge of their structure and synthesis pathways is required for the use of these LAB as antioxidants. Wild or cultivated mushrooms are rich sources of glutathione and ergothioneine, both having a specific role in defending mitochondria from oxidative damage related to the generation of ^•^O_2_^−^ due to the leakage of electrons during the mitochondrial ETS (electron transport chain). The presence of torularhodin, peroxiredoxins and thioredoxins in *Saccharomyces* sp. opens new paths for bioprospecting these compounds since yeasts are used as dietary supplements in the food industry and also have many other industrial applications. The mode of action of these microbial antioxidants has not been entirely elucidated, hence more in-depth studies are needed to improve knowledge and optimize their use. This might also lead to the development of new drugs and applications, taking advantage of the great biodiversity of nature.

## Figures and Tables

**Table 1:** Natural microbial antioxidant biomolecules with their chemical nature and bioactivity (Structures are retrieved from [29]).

Biomolecule	Microorganism	Structure	Chemical Nature	Molecular Weight	Chemical Formulae	Bioactivity	References
**Bacteria**							
**Astaxanthin**	*Agrobacterium aurantiacum, Paracoccus carotinifaciens*	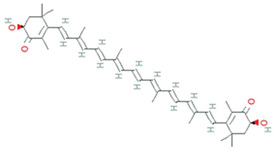	carotenoid	596.8 g/mol	C_40_H_52_O_4_	ROS/RNS, single- and 2-electron oxidants quencher, scavenger of free radicals	[30]
**Canthaxanthin**	*Bradyrhizobium sp. Lactobacillus pluvalis*	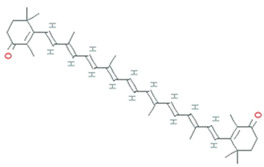	terpenoids	564.8 g/mol	C_40_H_52_O_2_	*increased resistance to lipid peroxidation*	[31]
**Phycocyanin**	*Pseudomonas sp.*	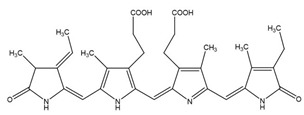	phycobiliprotein	30,000 Da	C_165_H_185_N_20_O_30_	Cytotoxicity, Neutrophil apoptosis	[32]
**Zeaxanthin**	*Staphylococcus aureus, Flavobacterium sp., Paracoccus zeaxanthinifaciens, Sphingobacterium multivorum*	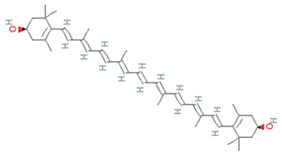	dihydroxy-carotenoid	568.88 Da	C_40_H_56_O_2_	protect cell membranes against oxidative damage	[33]
**Violacein**	*Janthinobacterium lividum, Pseudoalteromonas tunicate, Pseudoalteromonas sp., Chromobacterium violaceum*	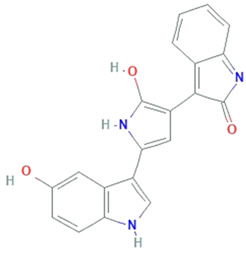	alkaloid	343.3 g/mol	C_20_H_13_N_3_O_3_	Protect against lipid peroxidation	[34]
**Granadaene**	*Streptococcus agalactiae*	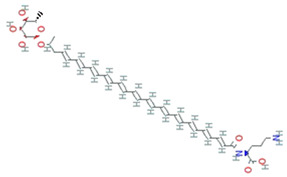	isoprenoid polyene	676.8g/mol	C_39_H_52_N_2_O_8_	ROS detoxification	[35]
**Staphyloxanthin**	*Staphylococcus aureus*	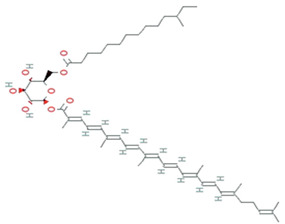	carotenoid	819.2 g/mol	C_51_H_78_O_8_	ROS detoxification	[36]
**Cyanobacteria**							
**Phycocyanin**	*Geitlerinema sp. TRV57, spirulina platensis*	-	phycobiliprotein	30,000 Da	C_165_H_185_N_20_O_30_	H_2_O_2_ scavenging activity	[37,38]
**Fungi**							
**Canthaxanthin**	*Monascus sp.*	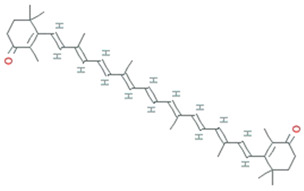	carotenoid	564.86 g/mol	C_40_H_52_O_2_	induction of catalase and superoxide dismutase	[31]
**β-carotene**	*Blakeslea trispora, Fusarium sporotrichioides, Mucor, circinelloides, Neurospora crassa, Phycomyces, Blakesleeanus*	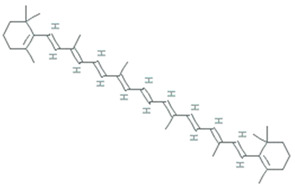	carotenoid	536.888 g/mol	C_40_H_56_	Antioxidant	[39]
**Lycopene**	*Fusarium Sporotrichioides, Blakeslea trispora*	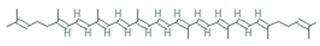	carotenoid	536.888 g/mol	C_40_H_56_	singlet oxygen-quencher	[40]
**Azaphilones**	*Talaromyces atroroseus, Penicillium purpurogenum*	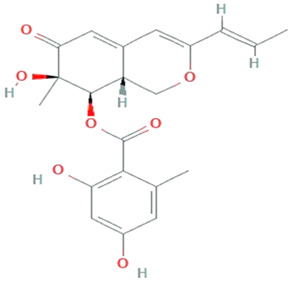	Polyketides	386.4 g/mol	C_21_H_22_O_7_	Activate PKS pathway	[41]
**Xanthomonadin**	*Xanthomonas oryzae*	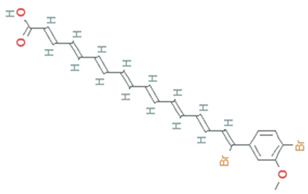	aryl polyene	518.2 g/mol	C_24_H_22_Br_2_O_3_	photoprotective activity against singlet oxygen analogous	[42]
**Riboflavin**	*Ashbya gossypi*	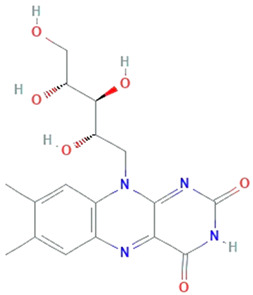	7,8-dimethyl-10-(1Υ-D-ribityl) isoalloxazine	382.32 g/mol	C_17_H_20_N_4_O_6_	Inhibit lipid peroxidation	[43]
**Endophytic fungi**							
**Chaetopyranin**	*chaetomium globosum, Microsporum sp., Epicoccum sp.*	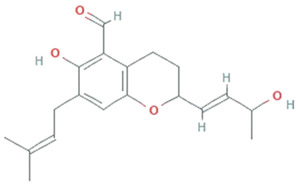	Benzaldehyde	316.167459 g/mol	C_19_H_24_O_4_	ROS scavanger	[44]
**Flavipin**	*Chaetomimum sp., Epicoccum nigrum, Aspergillus flavipes and Aspergillus terrus*	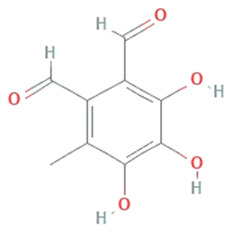	3,4,5-Trihydroxy-6-methyl-1,2-benzenedicarbaldehyde;1,2-Benzenedicarboxaldehyde, 3,4,5-trihydroxy-6-methyl	196.16 g/mol	C_9_H_8_O_5_	Inhibition of microsomal lipid peroxide	[45]
**Isopestacin**	*Pestalotiopsis microspora*	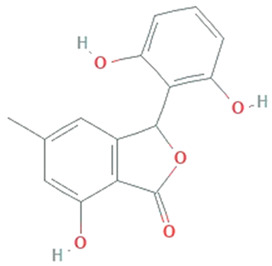	Isobenzofuranone	272.25 g/mol	C_15_H_12_O_5_	scavenge both superoxide and hydroxy free radicals	[46]
**Pestacin**	*Pestalotiopsis microspora*	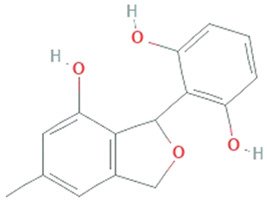	1,3-dihydro isobenzofuran	258.27 g/mol	C_15_H_14_O_4_	ROS scavenger	[46]
**Kaempferol**	*Mucor fragilis, Sinopodophyllum hexandrum*	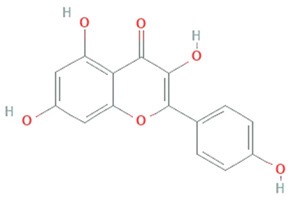	Tetrahydroxy flavone	286.24 g/mol	C_15_H_10_O_6_	ROS scavenger	[47,48]
**Tyrosol**	*Diaporthe helianthin, Glomerella cingulata*	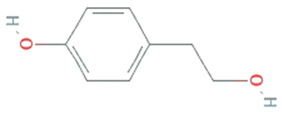	Phenylated alcohol	138.164 g/mol	C_8_H_10_O_2_	scavenging ROS and RNS	[49]
**Yeast**							
**Melanin**	*Saccharomyces, Neoformans, Candida albicans, Cryptococcus rajasthanensis*	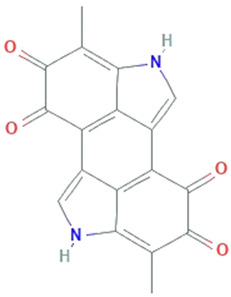	Natural cutaneous pigment	318.288 g/mol	C_18_H_10_N_2_O_4_	free radical scavenging activity and electron transferring properties	[50]
**Astaxanthin**	*Xanthophyllomyces dendrorhous, formerly Phaffia rhodozyma*	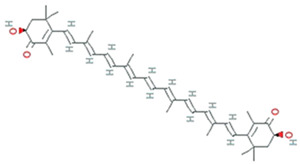	carotenoid	596.8 g/mol	C_40_H_52_O_4_	ROS/RNS, single- and 2-electron oxidants quencher, scavenger of free radicals	
**Microalgae**							
**Astaxanthin**	*Haematococcus pluvialis, Acutodesmus obliquus and Coelastrum sp.*	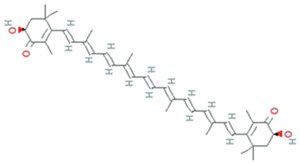	carotenoid	596.8 g/mol	C_40_H_52_O_4_	ROS scavenging and anti-inflammatory properties	[51]
**β-carotene**	*Dunaliella Salina, Tetraselmis sp. CTP4, Tetradesmus obliquus*	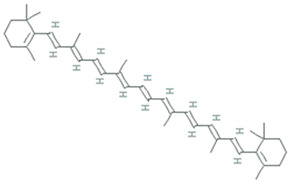		536.888 g/mol	C_40_H_56_	Scavenger of lipophilic radicals, inhibits lipid peroxidation	[52]
**Phycoerythrin**	*Porphyridium marinum, P. cruentum and P. purpureum*	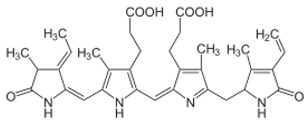	Natural light-harvesting pigment	~240,000 Da	-	Activate nuclear factor erythroid 2-related factor 2 (Nrf2)- Superoxide dismutases (SODs) pathways	[53]
**Lutein**	*Chlorella sorokiniana, Chlamydomonas sp.*	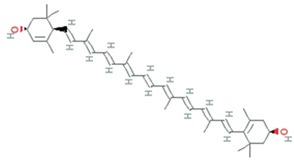	carotenoid	568.9g/mol	C_40_H_56_O_2_	block blue-light damage and quencher oxygen free radicals	[54]
**Phycocyanin**	*Spirulina platensis, Arthrospira platensis*	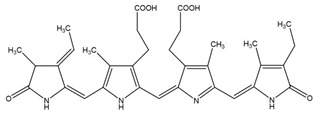	phycobiliproteins	~30,000 Da	C_165_H_185_N_20_O_30_	Quench ROS	[55]

**Table 2:** Characterization of bacterioruberin, and its most abundant derivatives.

Common Name	Molecular Formulae	Scientific Name	Producers	Mode of Action	References
**Bacterioruberin (BR)**	C_50_H_76_O_4_	2,2′-bis (3- hydroxy-3-methylbutyl)-3,4,3′,4′-tetradehydro-1,2,1′,2′-tetrahydro-γ,γ-carotene-1,1′-diol	*Haloarcula japonica*, *Halobacterium salinarum, Halorubrum sodomense* and *Haloarcula vallimortis* and *Halorubrum* sp. TBZ126	Protection against oxidative stress by arachidonic acid and H_2_O_2_	[71,72]
**Mono-anhydrobacterioruberin (MABR)**	C_50_H_74_O_3_	30-(2-hydroxypropan-2-yl)-2,6,10,14,19,23,27,33-octamethyl3-(3-methylbut-2-en-1-yl) tetratriaconta 4,6,8,10,12,14,16,18,20,22,24,26,28-tridecaene-2,33-diol	*Haloferax volcanii*	ROS scavenging activity	[71,72]
**Bis-anhydrobacterioruberin (BABR)**	C_50_H_72_O_2_	2,6,10,14,19,23,27,31-octamethyl3,30-bis (3-methylbut-2- en-1-yl)dotriaconta 4, 6, 8, 10, 12,14,16,18,20,22,24,26,28- tridecaene-2,31-diol	*Haloferax volcanii*	ROS scavenging activity	[72]

**Table 3:** Some antioxidant probiotic bacteria with the signaling pathways used to activate antioxidant enzymes and molecules.

Probiotic Bacteria	Pathway	Model Used	Effects	References
Probiotic Formulation SLAB51, *Bacillus*. *Longum*, *Lactobacillus acidophilus*	Sirtuin-1 (SIRT1)	Alzheimer’s disease (AD) (3xTg-AD) mice model	Increases antioxidant enzymes activity	[99]
*L. plantarum* LP6 *L. plantarum* C88 *L. rhamnosus* GG	PKC (protein kinase C)	Caco-2 cells Caco-2 cell Caco-2 cell	Enhanced activities of SOD and CAT Inhibit malondialdehyde (MDA) formation, raised SOD Ameliorate the oxidative stress-induced disruption of intestinal epithelial tight junction	[100,101] [102]
*L. brevis* SBC8803	Integrin–p38 MAPK (mitogen activated protein kinase)	Caco2/BBE cells	Induce cytoprotective heat shock proteins (HSP27) maintaining intestinal homeostasis	[103]
*L. plantarum*, *L. rhamnosus*, *Bacillus subtilis* JH642	p38 MAPK	RAW 264.7 cells Caco2_bbe_ cells	Decreases p38, JNK, ERK1/2 phosphorylation Increases p38 phosphorylation	[104] [105]
*Bacillus amyloliquefaciens* SC06 *Clostridium butyricum* MIYAIRI 588 *L. Plantarum* FC255	Nrf2-Keap1-ARE	IPEC-1 cell line rat smice	Increase CAT and glutathione S-transferase (GST) expressions	[106]
			Activation of ARE-dependent genes i.e., *GSTs*, and *TRX* Elevate the activities of SOD and GP_X_	[107]
				[108]
*Lactobacillus caseiBacillus* LBP32 *L. plantarum* CLP-0611	NF-κB	mice RAW 264.7 macrophages mice	Reduce cytokine production Reduce LPS-induced intracellular ROS accumulation Induced expression of M2 macrophage	[109] [110] [111]

## Data Availability

Not applicable.
